# Antitumor effector B cells directly kill tumor cells involving the CXCL12/CXCR4 pathway and their therapeutic efficacy is enhanced by IL-2

**DOI:** 10.1186/2051-1426-3-S2-P27

**Published:** 2015-11-04

**Authors:** Qiao Li, Yang Xia

**Affiliations:** 1 University of Michigan Medical Center, Ann Arbor, MI, USA; 2 University of Michigan, Ann Arbor, MI, USA

## Background

In cancer immunotherapy, effector B cells can inhibit spontaneous tumor metastases as well as local tumor growth in animal models. We have previously reported that anti-tumor effector B cells generated from tumor-draining lymph node (TDLN) could directly kill tumor cells *via* the Fas/FasL pathway and are regulated by IL-10. As a result, adoptive transfer of TDLN B cells conferred tumor regression in a spontaneous pulmonary metastasis mouse model of breast cancer, 4T1. Removal of IL-10, either by using IL-10^-/-^ TDLN B cells or by systemic neutralization of IL-10 using anti-IL-10 antibody, significantly augmented the therapeutic efficacy of adoptively transferred TDLN B cells.

## Methods

The 4T1 cell line is a mammary carcinoma syngeneic to BALB/c mice. 4T1 draining inguinal lymph nodes were collected and cell suspension prepared. CD19^+^ B cells were purified from the TDLN cells using MACS separator and were activated with LPS plus anti-CD40 mAb ascites. Cell surface expression of CD19 and CXCR4 and CD25 were analyzed by immunofluorescence assay. Healthy BALB/c mice were inoculated with 4T1 by s.c. 14 days after tumor inoculation, the mice were treated with tail vein injection of activated 4T1 TDLN B cells with or without IL-2 administration. 14 days after B cell transfer, all mice were sacrificed, and lungs were harvested for enumeration of spontaneous pulmonary metastatic nodules. The significance of differences in numbers of metastatic nodules, and cell lysis was determined using one-way analysis of variance (Newman-Keuls post hoc test) or unpaired Student's t-test.

## Results

Tumor cell death mediated by effector B cells involves the CXCR4/CXCL12 as well as Fas/FasL interactions. Blockade of CXCR4 and FasL using a CXCR4-specific inhibitor, AMD3100, and anti-FasL antibody, respectively, reduced the killing of 4T1 tumor cells by 4T1 TDLN B cells. Of note, blockade of CXCR4 and FasL concurrently inhibited B cell-mediated direct killing of tumor cells in an additive fashion, suggesting that both CXCL12/CXCR4 and Fas/FasL pathways are involved in the direct killing of 4T1 tumor cells by 4T1 TDLN B cells. In other experiments, we found that TDLN B cells express IL-2 receptors (IL-2R) and the ability of the TDLN B cells to inhibit metastasis was significantly enhanced with exogenous IL-2 administration.

## Conclusions

Besides our previously reported Fas/FasL pathway and IL-10-mediated suppression, our recently identified CXCL12/CXCR4 pathway and IL-2-mediated augmentation of the biological function of effector B cells suggests these B cells may represent an important component of the host immune response against tumor.

